# The Human Brain; Its Structure, Physiology, and Diseases

**Published:** 1847-10-01

**Authors:** 


### MEDICO-CHIRURGICAL

### REVIEW.

OCTOBER 1847.

### The Human Brain; its Structure, Physiology, and Diseases. With a Description of the Typical Forms of Brain in the Animal Kingdom. By *Samuel Solly*, F.R.S., Senior Assistant-Surgeon to St. Thomas's Hospital, and Lecturer on Clinical Surgery, &c. &c. Second Edition, with numerous Wood Engravings. 8vo. pp. 684. London: Longman, Brown, Green, and Longmans, 1847.

We had occasion, at no very distant period, to call the attention of our readers to the existing state of knowledge in reference to the Nervous System (see Med.-Chir. Review, July 1845). The several publications which were then noticed, valuable as they undoubtedly are, have by no means precluded the necessity for further and more varied cultivation of this deeply interesting branch of science; a consideration which causes us to hail with much satisfaction the re-appearance of a well-known and successful labourer in the field of neurological research. It is now some years since the first edition of Mr. Solly's work was published; and it is but just to this gentleman to state, that his treatise on the Brain was the first which, in this country, combined all the elements requisite for a scientific investigation; that is to say, in which comparative anatomy, embryology, experiment and pathology were brought to bear upon the anatomy and physiology of the human cerebrum. This enlightened mode of procedure is deserving of all commendation; and cannot fail, when generally applied, in stamping a philosophic character on Medical literature. In the present edition, the author has pursued the same course, and, by omitting some details of structure, in themselves of no great moment, he has been enabled without objectionally enlarging the work, to present the medical public with the most complete account of the anatomy, physiology, and pathology of the brain that has hitherto appeared. Mr. Solly has enriched the descriptive portion of the volume with a large number of wood-engravings, many of them the product of his own pencil; and which, being intercalated with the text, give an increased interest and clearness to the details they so admirably illustrate.

It is satisfactory to us to perceive that a writer so well qualified to pronounce an opinion as the author of this work, has fully recognised and ably supported those principles which, although they have been occasionally lost sight of or even directly opposed, we have ever regarded as the clue by which alone the complex organization and intricate actions of the nervous system can be successfully studied and interpreted. Two of the most fundamental of these principles are thus stated :—

"The revelations of the microscope regarding the ultimate texture of these different kinds of neurine are most deeply interesting, and quite determine the correctness of the view advocated in the first edition of this work, of their relative function. This view of the subject is now almost universally admitted, but in the year 1836 it was by no means an established point in physiology. The view to which I refer is this: that the cineritious neurine is the source of power, and the medullary neurine merely the conductor of it. The importance of establishing this position will be best understood when we come to the dissection of the human brain and spinal cord, and endeavour to discover the office of their component parts. Until this point was established, (and even now it is not considered to be so by all,) the study of the anatomy of the brain was barren and fruitless." P. 2.

A third fact, equally essential, is the strict independence, structurally and functionally, of the primitive nervous fibrillæ—"nerve-tubes never branch like blood-vessels and never inosculate with one another;" and, consequently, a "nerve-tube always performs one and the same office; it always conducts in the same direction, and the same kind of nervous power; not at one time carrying impressions which, on reaching the brain, become sensations, and at another time conveying orders to a muscle to contract." (P. 12.) The power of the primitive fibrillæ being thus limited, the varied actions of the nervous system necessitate the provision of distinct classes of conductors, which the author thus, correctly as we conceive, sets forth :—

"We find tubular neurine performing various offices :—

"1st. As a conductor of an impression from the surface of the body to the brain,—a *nerve of sensation.*

"2ndly. As a conductor of an order to act, from the brain to the voluntary muscles—*nerve of volition.*

"3rdly and 4thly. As a conductor of an impression from the surface of the body to the spinal cord, which is reflected thence down another set of conductors to the muscles whereby they are called into action, independently of volition; —the *excito-motory nerves* of Dr. M. Hall.

"5thly. As a conducting medium between the centres of power—the *commissures.*" P. 7.

Mr. Solly assumes, as the basis of all inquiries into the nervous centres of man, the facts revealed by comparative anatomy. In the Preface we find these judicious remarks: "The only philosophical method of simplifying and giving a character of general interest to the anatomy of the human brain, is by commencing with the structure and functions of a nervous system in the lowest and simplest forms of animal existence, rising by degrees to the highest, carefully observing each addition of parts, and the relationship borne by these to an addition of function. By pursuing this course we shall be rewarded by finding that the encephalon, this apparently most complicated organ in the human being, is but a gradual development from an extremely simple fundamental type on one uniform and harmonious plan, and that the seeming complexity of the cerebro-spinal axis in man really arises from the great concentration, as opposed to the extreme diffusion of its component parts in the lower order of animals; for in no particular are the higher orders more strikingly distinguished from the lower than in the concentration of function within circumscribed spaces." (*Preface*, p. x.) In interpreting the evidence derivable from this source, the author applies as a realised and fertile principle this fact—that "one of the most important functions of a nervous system, as regards the vital existence of an animal, is to receive impressions, and to re-act on such impressions, independent of the consciousness or the will of the individual: this fact will be found universal in its application." In other words, the author unreservedly adopts the views of Marshall Hall on the physiology of the nervous system; and those of Grainger, Carpenter, and Newport on its anatomy.

We must pass by, though reluctantly, the details connected with the invertebrate animals, and proceed briefly to notice the interesting facts illustrative of the conformation of the brain among the vertebrata. In order to seize upon the fundamental organs, in the midst of the intricacies of apparent confusion of the cerebral parts, the author assumes three leading facts:—1, that every nerve of sense, whether it be of the sense of smell, sight, hearing, taste, tact, or of simple sensation, has, at its central extremity, a collection of cineritious neurine, or a ganglion; 2, that there is an organ for directing and controlling the actions of locomotion, corresponding to the cerebellum; 3, that there are parts, analogous to the hemispheres of the human brain, which constitute the instrument of the mental operations. We have no doubt that, so far as the anatomy is concerned, this is the true principle in accordance with which the encephalon must be studied—that it is the only mode of escaping from that obscurity, in which, as Mr. Solly justly observes, writers on comparative anatomy have involved this deeply interesting subject. As it is quite clear that the brain of fishes must serve as the basis for the comparison of the higher vertebrata and man, it becomes a point of primary importance to fix upon the signification of the curious series of ganglia which are seen on exposing the cranial cavity in some of the simplest animals of this class. There is one point connected with this inquiry, which, if not truly determined, throws the whole subject into inextricable confusion—we refer to the just appreciation of the *olfactory ganglia*. These bodies, whether placed close to the hemispherical ganglia, as in the eel, or removed from them to a considerable distance, as in the whiting, constitute invariably the most anterior pair of the encephalic masses. To the author belongs the merit of having given the true homology of these ganglia more than ten years ago, when the most eminent authorities, among whom we may adduce Serres, had evidently no clear idea of their signification. Placed next in order behind the olfactory ganglia or lobes, are the two hemispherical ganglia, as Mr. Solly proposes to call the hemispheres; then follow the two optic ganglia; and, lastly, the cerebellum. There are several other subordinate masses, for an account of which we must refer our readers to the work before us, and to the Hunterian Lectures of Professor Owen. Thus, among fishes, are to be recognised the typical elements of the human brain, the vast distance between them being filled up by the multitudinous forms displayed in the ascending animal scale, and by the no less exact evidences of developmental anatomy.

We regret that our limits will not permit us to follow the author through his clear and comprehensive account of the successive perfectioning of the cerebral organization ; and especially of the laws in obedience to which the convolutions are developed. The whole of this part of the work, illustrated as it is with a great number of admirably executed woodcuts, is deserving of careful perusal, and will, we feel assured, receive general commendation. Before quitting this subject of the comparative anatomy, it may, however, be desirable to extract the following passage, in which several errors are corrected respecting the existence of that interesting structure, the *rete mirabile,* a rich network of vessels into which the carotid artery divides after it has penetrated into the skull, and lies beneath the dura mater by the side of the sella turcica.

"Rapp found this plexus in the stag, roe, the fallow-deer, chamois, the goat, sheep, and calf, and oxen. He considers that the arrangement of the foramina in the base of the skull in the camel indicates its existence in this animal, but he has not had the opportunity of seeing the parts in a recent state. It exists also in swine, but it does not occur in other Mammalia besides the Ruminantia and swine. Cuvier's statement certainly differs from this : he says that this vascular arrangement appears to occur in most of the Carnivora, but is absent in the elephant and beaver. According to Carus it is present in most Mammalia, and Willis says it exists in the dog, the fox, cat, &c. ; but this is a mistake, for it does *not* occur in the dog, fox, badger, weazel, otter, or hedgehog, or in the domestic cat. But it has been found by Mr. Quekett in the leopard. Neither is it found in man, the apes, horse, elephant, or the Rodentia." P. 137.

It is curious that in that remarkable ruminant the Giraffe, notwithstanding it feeds on the tops of trees and with the head therefore elevated, there is a well-developed rete mirabile, a fact which has been lately ascertained in opposition to the commonly-received opinion, by Mr. Quekett ; and which proves, what is shown by so many other instances, that unity of structure is a more predominating law in the animal kingdom, than that of final causes or design.

In the Section on the Spinal Cord, Mr. Solly expresses his conviction of the accuracy of Mr. Grainger's account of the origin of the nerves ; and he has given a most interesting figure of a minute dissection, made with the assistance of that gentleman, in which the fibres of the anterior root are represented as becoming partly continuous with the longitudinal fibres of the spinal marrow, and partly as running into the gray matter of the cord and there terminating. These results correspond with those obtained by Dr. Budge, noticed already in this Journal (Med.-Chir. Rev., 1845, p. 14) ; and, as both examinations appear to have been made with much care by the aid of the microscope, they may be received as correct.

The author has given a most minute and detailed account of the composition of the medulla oblongata ; of the signification of the various nuclei of gray matter there observed ; and especially of the distribution and physiological endowments of the several fasciculi or strands of white fibres connected with this vital segment of the nervous centres. Although some of our readers may suppose that these anatomical intricacies concern rather the student than the practitioner, they will, on well considering these details and on scrutinising the excellent figures by which they are brought before the eye, find that a knowledge of this complex organization is indispensable to the satisfactory investigation of cerebral pathology. The reason why the descriptions of the anatomist have, as regards this part of the body, become so distasteful, is because they have been unfruitful; because, in the midst of a detail which is discouraging and redundant even to professed teachers, not a glimpse of those relations upon which the actions of the brain, whether healthy or morbid, repose, is to be caught; because, in fine, the authors of France and Germany, and herein too often imitated by our own countrymen, have broken up the brain into an infinitude of fragments, insignificant in themselves and utterly obstructive to those general and connected views of structure, which can alone subserve an enlightened physiology. The account given by Mr. Solly is opposed in toto to this barren topographical school: the several parts, instead of being considered piece-meal, are invariably viewed in reference to those other structures with which they are allied as members of an active living organization: if complex bundles of fibres are to be considered, the tedium of tracing them is obviated, by making the anatomical process reveal some interesting physiological action; or, if nodules of the vesicular matter are to be decribed, the question is not what is the precise shade of colour or the exact number of indentations, but what part does it play in the mysterious operations of conscious existence.

But we must allow the author to speak in his own language.

"According to the plan generally pursued in treating on the brain in systematic works of anatomy, the information conveyed amounts to little more than a vain catalogue of names applied to parts, without reference to their structure, their functions, or even their analogies in the nervous system of the lower orders of animals. Such a barren prospect as a list of names holds out but little to attract the most zealous among students, while the dryness of unconnected detail, and the obstacles to clear conceptions engendered by the absence of everything like arrangement, almost certainly deter him from attempting to learn more than is required to prepare him for examination for the diploma. It is unfortunate, indeed, that candidates for this honourable certificate are still very generally required to describe the appearances presented by the brain dissected, or rather destroyed, by the old method of slicing; a method most unphilosophical in its conception, and totally inadequate to impart any real information in regard to the structure of the organ. And I do not hesitate to affirm that this mode of examination has contributed essentially to retard the diffusion of sound knowledge in regard to the anatomy and physiology of the most important system in the body."—*Preface*, p. vi.

We had hoped that the mode of examination here so properly condemned, had been long since abolished: if, however, any remnants of the old and barbarous system still linger in high places, we trust the scientific works on the brain, which have lately issued from the press of this country, will speedily induce the examining bodies to place themselves on a level with the present state of knowledge.

A few extracts will serve to justify the very favourable opinion we have expressed of Mr. Solly's work; and, also, to illustrate what we conceive to be the true method of pursuing the anatomy of the nervous centres. The question respecting that somewhat unpromising subject—the fissures and subdivisions of the spinal cord, is thus discussed :—

"*Line of demarcation between the tracts of sensation and motion.*—Although the different offices performed by the anterior and posterior roots of the spinal nerves have been, I think, clearly ascertained, and as it is also evident that the spinal cord consists of tracts of neurine whose office is the same as the nerves which are connected with them, and therefore that there are portions of the cord which perform functions as distinct from each other as the arteries and veins, still anatomists are not yet agreed as to the line of demarcation between them. Sir Charles Bell, for instance, in a paper published in the 135th vol. of the Philosophical Transactions, states that he regards the lateral portion of the antero-lateral columns as a part of the tract for sensation, and I have no doubt of its correctness. The circumstance of there being no decided anatomical line of division between the two columns is not of itself an argument against the correctness of this view; for it is quite possible that perfect distinctness of parts, as regards their function, without any visible line of separation between them, may exist. We must always bear in mind that the neurine which composes the cord is supported and clothed by a perfect though delicate membrane, which, pervading its substance in every direction, is undoubtedly as capable of separating masses of neurine endowed with distinct powers, and ordained by nature to execute distinct offices from each other, as any fissure however wide, or membrane however thick. The presence of such gross and palpable partitions, it is true, would save us some trouble in discovering the line of demarcation, but would not necessarily make it in any way more efficient. They are not the less distinct organs because of our ignorance of their respective limits, any more than a nerve of motion is one of sensation because we are incapable of unraveling the fibres of each from their common investing membrane.

"That the boundary line between the two organs of sensation and voluntary motion comprised within the spinal cord cannot be formed by the posterior peak of grey matter, is very decidedly proved by the fact that a portion of the fifth pair of nerves, which we know to be a nerve of sensation from the beautiful experiments of Mayo and Sir Charles Bell, is not connected with the posterior, but with the lateral columns."—*L. c.* p. 223.

This is as it should be; the physiologist refusing to be bound by the apparent evidence of anatomy, when that evidence is over-ruled by superior considerations; but availing himself of a significant structural arrangement, to explain a difficult point of function. We are reminded by this quotation, that, as far as our memory serves us, the merit of seizing upon the precise attachment of the portio major of the fifth pair, as an indisputable indication of the extent of the sensory column, in this and other parts of the work, is due to the author.

There is no point of greater interest to the practitioner, than to have a clear conception of the mode in which the cerebellum and the cerebrum are respectively brought into connexion with the motor and sentient organs of the animal frame; for without this knowledge no satisfactory investigation can be made into a single case of paralysis, apoplexy, or convulsion. There is one fundamental truth which is the sole clue in all these cases; and which, although it may appear to be sometimes contravened by peculiar and inexplicable symptoms, is nevertheless recognisable on a careful scrutiny as a real principle in cerebral pathology: it is this—that the morbid effects, as evidenced by disturbance of the organs of sense and motion, are to be understood by following the tracts of fibrous matter proceeding from the spinal cord to the cerebrum and cerebellum. In this point of view, the physiological character of the fibres composing respectively the corpus restiforme going to the cerebellum and the crus cerebri running to the cerebrum is a point of primary importance. The credit of explaining the true constitution of the former of these cords belongs exclusively to Mr. Solly; for although some preceding writers, and especially Rolando, were in part acquainted with the fact that some fibres of the anterior cords of the medulla spinalis passed towards the cerebellum, the author was the first anatomist who discovered that the corpus restiforme receives fibres from the anterior or motor column of the spinal cord. He remarks, with justice, that "the fibres just described as connecting the antero-lateral columns of the cord with the cerebellum are peculiarly interesting when viewed in relation to the functions of the cerebellum. For although it is true that its functions have not yet been clearly ascertained, the experiments of Flourens, Bouillaud, Magendie, and others, and the numerous cases on record in which disease of the cerebellum has been followed by paralysis, all tend to prove that the cerebellum is in some way or other connected with the regulation of muscular action, most probably, as before hinted at, that it has the power of combining the action of individual muscles so as to effect an harmonious result, such as is necessary to enable us to stand, walk, &c."—*L. c.* 231.

Our readers will agree in the truth of these observations, when they recollect that some most distinguished authorities, being ignorant of this connexion between the lesser brain and the motor tract of the cord, and equally uninformed as to the true formation of the crus cerebri, have not hesitated to enunciate theories, which, if received, would involve the whole pathology of the encephalon in hopeless confusion. Thus, at no very distant period Foville, assuming that the posterior columns of the spinal medulla are alone prolonged into the cerebellum, and the anterior or motor as exclusively into the cerebrum, inferred that the former is more especially the organ presiding over the sentient phenomena, and the latter, or cerebrum, as exclusively the agent predominating over voluntary motion; an hypothesis which, notwithstanding it was opposed to all the evidences of physiology and pathology, received a considerable portion of assent, and was even admitted as a probable opinion by that eminent writer Dr. Pritchard.

As in addition to the motorial fibres, the corpus restiforme is well known to receive threads from the posterior aspect of the spinal cord, or the sentient tract; and as moreover there is evidence to show that the restiforme fibres form a part of the decussating apparatus of the medulla oblongata, all the anatomical relations required to explain the phenomena of disease are realised. The subject thus elucidated, there is no longer any difficulty in comprehending how irritation of the cerebellum may disturb the muscular actions; how effusion of blood into its substance may cause a paralysis both of motion and sensation; and, lastly, as so many cases, especially those recorded by Andral prove, how extravasation or ramollisement of one of the hemispheres will, as in the case of the cerebrum, induce hemiplegia on the *opposite* side of the body. A reference first of all to the graphic illustrations of Mr. Solly (especially to figs. 88, 89 and 90), and then to the sections on the pathology of the cerebellum, will remove all obscurity on this very interesting and difficult subject.

The true anatomical structure of the crus cerebri is a point of equal importance with that of the restiforme body; as this, however, is so well known, we will only call attention to the circumstance of there being, altogether distinct from the decussation of the pyramidal bodies, a distinct decussation of the fibres forming the sensory tract of the cerebrum. This interesting disposition takes place where these sensory tracts form the floor of the iter a tertio ad quartum ventriculum ; and, as many anatomists have overlooked its existence, the following account of the mode of exposing this structure may not be unacceptable :

"The best mode of demonstrating this interlacement is first to separate the medulla oblongata, with the pons Varolii, crura cerebri, and optic thalami, from the rest of the brain. Secondly, divide the pons Varolii anteriorly, in a longitudinal direction, through the centre to the depth of half an inch; divide the pyramidal decussation; then take the two lateral halves of the cord and split them upwards, tearing through the floor of the fourth ventricle. When the rent passes the roots of the auditory nerve, fibres, the size of ordinary ligature silk, may be seen running obliquely across the mesial fissure, from one side to the other, decussating with their fellows. This decussation may also be demonstrated anteriorly, though it requires more care and some dissection." P. 243.

We need hardly observe that this intercrossing of the sentient fibres, regarded in connexion with the decussation of the motor tracts or corpora pyramidalia, offers a perfectly satisfactory explanation, why, in cases of effusion into one hemisphere, sensation as well as motion, is lost on the opposite side of the body.

As it is one principal object with us to lay before our readers, with the assistance of the work before us, some of the fundamental facts connected with the organization of the nervous centres, we would now call their attention to two points, which, although they are perhaps of greater importance than any others relating to cerebral anatomy, have been either denied or doubted by some late writers, and especially by Foville. The first of these questions concerns the ultimate destination of the motor and sentient fibres of the crus cerebri. That these fibres, after traversing the corpus striatum and optic thalamus, continue their course and run finally into the very substance of the cerebral convolutions, is one of the most fertile truths for which anatomical science is indebted to Gall and Spurzheim, but especially to the former. Mr. Solly has always contended for the correctness of this view of the subject; and, as it affords a satisfactory explanation of some of the most common cases of cerebral disease, we cannot do better than follow the example of our author, who has quoted the following familiar but truthful description from the great founder of modern physiology, Sir Charles Bell.

"The thalamus forms a nucleus round which the corpus striatum bends, and when their respective layers of striæ make their exit beyond these bodies to form the great fan, or solar-like expansion into the hemisphere of the cerebrum, their rays mingle together. A rude representation of these two parts of the cerebrum as we have traced them may be made with the hands. If I place my wrists together, parallel, and closing one hand embrace it with the other, I represent the two portions of one crus. The closed fist is the thalamus, and the other is the corpus striatum. If I then extend my fingers, interlacing their points, I represent the final distribution of the portions of the nervous matter which are dedicated to sensation and volition." P. 244.

A second point, which has also been placed in some degree of doubt, is the real formation of the corpus callosum. If we are to adopt the hypothesis of Foville, this vast organ, the largest of the encephalon, is nothing else than a connecting medium between the crura cerebri, instead of being, what is shown equally by careful dissection and comparative anatomy, the great commissure of the hemispheres of the brain. The author, herein concurring with the best English anatomists, contends for the truth of the latter account;—" the great *transverse commissure,* or corpus callosum, is a body consisting of fibres of medullary neurine, the extremities of which are everywhere in contact with the internal or central surface of the cineritious layer which forms the convolutions of the hemispheres,—the hemispherical ganglia. These fibres consequently establish a communication between the cineritious neurine of the whole convoluted surface of both sides of the cerebrum."—(*L. c.,* p. 250.) This account is illustrated by an admirable and most correct figure of the fibrous expansion above described.

Condemning, as we totally do, the cumbrous descriptions still persisted in by so many writers and teachers; and confidently anticipating that, at no distant period, the whole of these will be replaced by an intelligible and simple anatomy of the brain, we have much pleasure in extracting the following judicious observations, with which the author concludes his philosophic account of that organ:

" In conclusion, let me express the hope that these views or analyses, if I may be allowed so to call them, of the component parts of the encephalon will really simplify the whole of its anatomy, and materially assist the student in acquiring a knowledge of its true character. I wish that custom did not require the student to burthen his memory with fanciful and unmeaning names, and that, instead of learning a long catalogue of the contents of the lateral ventricles as they are erroneously designated, and puzzling himself with the absurd titles of hippocampus major and minor, pes hippocampi, tænia hippocampi, cornu Ammonis, &c., he should be required simply to observe how the spinal columns appear to terminate superiorly in two large tubercles, the *corpora striata* and *thalami,* from the sides and under parts of which the hemispheres spring out, being afterwards reflected so as completely to envelope this bulbous extremity of the spinal cord. In the same way the third ventricle should be described as a fissure separating the two halves of the brain, his particular attention being directed to the commissures which pass across it to connect the different cerebral ganglia with one another. The description of the relative position of these ganglia, the commissures connecting them, and their relation to the ganglia and columns of the spinal cord, comprehend all the information which is either interesting or useful to the student." *L. c.* p. 283.

We regret that our limits compel us to pass by the section treating on the central attachments of the so-called cerebral nerves, and especially that relating to the development of the nervous system.

In the tenth chapter the author gives a brief resumé of his physiological views, in many of which we fully coincide; but from others we must withhold our assent. The following are regarded, and justly, as " fundamental principles."

" 1. That vesicular neurine is the source of power.

" 2. That medullary neurine is the conductor of it.

" 3. That medullary neurine is also the conductor of those impressions which call forth the power of the vesicular neurine.

" 4. That the vesicular neurine is collected in masses of variable form and size—the *ganglia.*

"5. That the medullary neurine is moulded into cords and bands—the *nerves* and *commissures.*" P. 329.

The functions of the nerves are thus described:—

"The experiments of Sir C. Bell, Magendie, and Mayo, have proved that there are nerves subservient to sensation—sensiferous or sensory nerves, and nerves of voluntary motion. The physiological researches of Whytt, Prochaska, and, more perfectly, Marshall Hall, confirmed by the anatomical observations of Grainger, Carpenter and Newport, have established another system of nerves for the involuntary—the conservative movements of the body under the title of the excito-motory system of nerves. All sound research and careful experiment prove that a nerve in the whole extent of its course, whether that course is between the fibres of a muscle, in the canal of a bone, in the substance of the spinal cord, in the crura of the brain, or in the masses of the hemispheres, always performs one and the same office, conducting always in one and the same direction." P. 330.

In approaching the complex organs enclosed within the skull, Mr. Solly is impressed with the same difficulties that have obstructed all physiologists labouring in the same field: vivisections, comparative anatomy, and pathology, have thrown much light upon the mysteries of the mental phenomena, but still the curious inquirer is but too often doomed to disappointment. The author is inclined to adopt a theory, already advocated more or less by several eminent writers, among whom may be enumerated Drs. Todd and Carpenter and Mr. Bowman, to the effect that certain masses of grey matter—the auditory ganglia of the medulla oblongata, the tubercula quadrigemina, the optic thalami, and the corpora striata, are, independently of the cerebral hemispheres, so many separate "sensorial centres," that is distinct organs of perception.

As there appears to us to be some obscurity, if not contradiction, in the views which Mr. Solly states he has adopted from Dr. Carpenter, we prefer presenting them to our readers in his own words.

"The anterior and posterior cerebral ganglia are regarded by Dr. Carpenter as forming part of the series of sensorial centres, of which we have seen other members in the olfactory, optic, and auditory ganglia. That they are independent centres of action, not mere appendages to the hemispheric ganglia, appears from the large quantity of vesicular neurine which they contain; and that the corpora striata are so, further appears from the absence of any correspondence in size between them and the hemispheric ganglia. Thus in fishes, we find that the corpora striata make up the principal bulk of the second pair of masses; in reptiles, birds, and the lower Mammalia, they still form a very large portion of that which is commonly termed the cerebrum; and their subordinate aspect in man and the higher Mammalia is solely due to the large relative development of the hemispheric ganglia. On the other hand, there is scarcely any rudiment of the thalami optici to be discovered in fishes; their proportional size increases in reptiles, birds, and the lower Mammalia; but it is only in man that their dimensions approach those of the corpora striata. The peculiar connection of the thalami optici with the posterior columns of the spinal cord, and their great development in man, suggests the idea that they are the ganglia of *tactual* sensation; whilst the connection of the corpora striata with the anterior columns indicates their relation with the motor function. The very close relation between the thalami optici and the corpora striata—corresponding, as Messrs. Todd and Bowman have suggested, with that which exists between the posterior and anterior peaks of grey matter in the spinal cord—harmonizes well with the fact that the greater number of muscular movements are directed by common sensation; whilst the special connection established by the inter-cerebral commissure between the corpora striata and the optic ganglia (tubercula quadrigemina) explains the peculiar influence of the sense of light in directing certain classes of muscular actions. The communication which is formed by the medullary substance of the cerebrum between these ganglia and the hemispheric ganglia seems to be the medium by which *sensations* are transmitted to the latter, to become the stimulus of intellectual operations, and by which the influence of *volition* is transmitted downwards to excite muscular motions through the corpora striata." P. 334.

In addition to these functions, the "whole chain of sensory ganglia is regarded as the centre of those *automatic* muscular movements which differ from those of a simply reflex character in being dependent upon sensation;" such as the instinctive actions of the lower animals, and a variety of actions performed by the human being—the consensual movements of the eyes, the regulation of the laryngeal muscles in the production of vocal sounds, the convulsive movements in hydrophobia, brought on by the sight or sound of water, &c. As to the hemispheres, they are the exclusive organ of the mental functions—the instrument by which the sensorial impressions are not only perceived, but are converted into ideas.

As we have already stated (Med.-Chir. Rev. 1845, p. 490), some of the reasons which may be adduced in opposition to these doctrines, we would only observe further that, up to the present time, they have not been reconciled, so far as they relate to the seat of consciousness, with certain familiar and admitted facts in physiology and pathology. One or two of these only can be now quoted: if both hemispheres be removed in a dog, as in Hertwig's experiments, the animal does not hear the report of a pistol; if the hemispheres be excised in a pigeon, sight and hearing are abolished; if effusion of blood take place into the substance of one hemisphere *above* the corpora striata and optic thalami, or if from fracture of the skull a portion of bone be driven into the same part, inducing softening and suppuration of the white fibrous matter, there is paralysis more or less complete of the opposite side of the body. Now in all these and similar instances the assumed "sensorial organs," "the whole chain of sensory ganglia," are left intact; and yet the phenomena of consciousness, attributed by this theory to them—vision—audition—tactual sensation, and voluntary motion, are destroyed. There is one fact connected with these cases, which is most significant; and which, whatever theory is adopted as to the organ of perception, must ever be regarded as the clue to the whole subject of paralysis and convulsion, as far as the brain is involved: we allude to the well-known circumstance that, in effusion and compression connected with one hemisphere, the paralysis is on the *opposite* side of the body. This is a clear demonstration that the decussating fibres already noticed, constitute, in reality, what physiologists contend they are, *isolated conductors;* for the mischief is traced precisely by following their remarkable and peculiar track. This fact, and the no less instructive cases of paralysis affecting special muscles of the orbit, further indicate that every affection of convulsion and palsy connected with the cranial nervous centres, however complex and apparently contradictory they may be, must be studied in strict reference to the course and destination of the primary fibrillæ. That the whole subject is still involved in much difficulty we are most ready to admit; but at the same time we must reiterate our conviction, that this will never be removed till physiologists, by returning to the doctrine formerly received, namely, that the cerebral hemispheres are the exclusive seat of all consciousness, shall place their conclusions in harmony with the unquestionable results of pathology. In treating of convulsive affections Mr. Solly quotes some extremely apposite remarks of Lallemande, to which, as bearing on the question just considered, we would call the attention of our readers (p. 554 *et seq.*)

Although we have ourselves no faith whatever in the truth of phrenology, it would be inconsistent with candour to withhold the author's arguments in favour of this popular doctrine.

"My reasons for believing that there must be a great deal of truth in phrenology are fourfold. First. I have received from practical phrenologists, and especially the late worthy Mr. Deville, such accurate characters of individuals known to me, but unknown to them, that I cannot believe the accounts I received could be the result of accident and conjecture, which must have been the case if phrenology is untrue.

"Secondly. Phrenology alone—as it appears to me—can account for all the varieties of insanity, especially monomania.

Thirdly. The facts which have been collected by the late Mr. Deville, showing that the brain will alter its form at any period of life.

"Fourthly. The existence of longitudinal commissures." *L. c.*, p. 339.

We need scarcely remark that great importance is attached by phrenologists to the evidence derived from the investigation of insanity. Upon this most interesting subject Mr. Solly observes:—

"If phrenology is true, insanity on its first ingress is frequently not a disease of the whole brain, but of only a part of it. The first effect of inflammation is to excite to an unnatural degree the natural function of an organ. The function of the organ thus exalted obtains a mastery over the rest. For instance, a man, from defective education, combined with hereditary tendency, allows his love of approbation, his vanity, in other words, to grow with his growth, and strengthen with his strength, gradually becoming the sole ruling principle of life: at last it, instead of reason, so completely guides and regulates all his actions, that they are contrary to reason, and justly called the acts of a lunatic. Yet all this may go on with reasoning faculties so acute, that he conceals the dominant feeling of his breast, the mainspring of all his actions, and in a court of law defies any one to prove him insane.

"The great amelioration which has been effected in the condition of the lunatic has been founded on this principle, that none are so mad as to be incapable of appreciating kindness. Throughout all the admirable and interesting reports of Dr. Conolly, it will be seen that this has been the guiding principle of his boldly humane treatment. The first thing, says this admirable man, is to gain the *confidence* of your patient; and that once obtained, you may do *anything* with him.

"Now if this is true, (and no one who has treated the insane on these principles doubts it,) so is it equally true that they may be awed by punishment and even acknowledge its justice. Only the last time I had the pleasure of visiting that noble asylum, Hanwell, I listened with much interest to a lunatic whom we met in the grounds. He began by requesting Dr. Conolly to procure his release from the Asylum, and then went on in a rambling manner, reasoning on things and circumstances which had no existence, showing his mental aberration; but he finished by saying, as an argument for his being allowed his liberty, *that he*

*had always conducted himself with propriety* while there, which was perfectly true. This sense of right and wrong was as perfect as ever, and this sense enabled him to conduct himself properly. But if he had supposed that the circumstance of his being lunatic gave him a license for any conduct, and freed him from all responsibility, would he have been so anxious to conduct himself properly? And if he were told that the law of the land would not take notice of an improper act, even if that act amounted to the murder of a fellow-creature, he would not feel the same reason for self-control." P. 340.

The portion of Mr. Solly's work which treats of the diseases of the brain, is distinguished by a happy combination of practical observation with scientific research; and as, in addition, the views of the most eminent modern writers are given in their own words, a comprehensive and valuable résumé of cerebral pathology as it is cultivated in the present day, is presented to the reader. In a subject so comprehensive, we can only select one or two of the more general topics for consideration. The observations of the author on derangements of the circulation, especially on the various forms of anæmic affection, are very judicious, and well worthy the attention of the practitioner. Those only who have carefully investigated the complex texture of the brain and spinal cord with the aid of the microscope, are capable of forming a just conception of the predominating influence that must be exerted on the actions of these organs by disturbance of their capillary system. Impressed with this conviction Mr. Solly, in considering the cause of the symptoms characteristic of cerebral anæmia, dissents from the opinion of Dr. Burrowes, that this consists of diminished pressure; he thinks, and in this view we concur, that the mischief arises from the defective supply of blood in the capillary vessels.

"We know that the function of all other organs, uninfluenced by pressure, may be excited by a flow of blood into them, or their function may be arrested by any stoppage in their supplies. Take the salivary glands or the testicles, as an illustration: mental emotions will both excite and arrest their secretions; and I believe that the brain would be similarly affected, and to the same extent as now, even if that organ were not enclosed in a spherical box, and supported on all sides by the cerebro-spinal fluid."—*L. c.*, p. 349.

In speaking of anæmic coma, the author enforces the necessity of well distinguishing between this affection and the coma arising from inflammation and effusion—a distinction of vast importance in practice, and for firmly establishing which the profession is mainly indebted to that admirable observer Dr. M. Hall. Although much attention has been paid to this class of affections, the following judicious observations of Dr. Gooch will not be here misplaced:—

"So inveterate is the disposition to attribute drowsiness in children to congestion of the brain, and to treat it so, that I have seen an infant, four months old, half dead from the diarrhœa produced by artificial food, and capable of being saved only by cordials, aromatics, and a breast of milk; but because it lay dozing on its nurse's lap, two leeches had been put on the temples, and this by a practitioner of more than average sense and knowledge. I took off the leeches, stopped the bleeding of the bites, and attempted nothing but to restrain the diarrhœa, and get in plenty of nature's nutriment, and as I succeeded in this the drowsiness went off and the child revived. If it could have reasoned and spoken, it would have told this practitioner how wrong he was; any one, who from long defect in the organs of nutrition is reduced so that he has neither flesh on his body nor blood in his veins, well knows what it is to lay down his head and dose away half the day without any congestion or inflammation of the brain. This error, although I have specified it only in a particular complaint of children, may be observed in our notions and treatment of other diseases, and at other periods of life. If a woman has a profuse hæmorrhage after delivery, she will probably have a distressing headache, with throbbing in the head, noises in the ears, a colourless complexion, and a quick, weak, often thrilling, pulse, all which symptoms are greatly increased by any exertion. I have seen this state treated in various ways, by small opiates, gentle aperients, and unstimulating nourishment, with no relief. I have seen blood taken away from the head, and it has afforded relief for a few hours, but then the headache, throbbing, and noises, have returned worse than ever; the truth is, that this is the acute state of what, in a minor degree, and in a more chronic form, occurs in chlorosis, by which I mean pale-faced amenorrhœa, whether at puberty or in after-life. It may be called acute chlorosis, and, like that disease, is best cured by steel, given at first in small doses, gradually increased, merely obviating constipation by aloetic aperients."—*L. c.*, p. 367.

The necessity of employing the microscope in the investigations of morbid anatomy, is strongly advocated by the author; indeed, when it is recollected that the essential structure of every organ in the body—in fact *the* organ physiologically considered, is altogether hid from the naked eye; that the capillary vessels, constituting the active part of the vascular system—the cells of the various glands, forming the real apparatus of secretion; the gray corpuscles and primary tubules composing the instrument of innervation, are each and all of microscopic dimensions, what reasonable expectation can there be of detecting those molecular changes, which constitute the first and essential alterations in all organic disease, so long as the examination is made by the unaided senses and the very objects implicated are unseen? In some instances, indeed, the microscope will do little more than disclose the real seat of the disease; but in others, it will also reveal important indications explanatory of the true character of the disease. One of these latter cases concerns that obscure affection, *ramollissement* or softening of the brain, a subject on which the author has collected much valuable information. Our readers will recall the involved and contradictory opinions of writers as to the true nature of this affection; the long discussions whether it be, as Lallemand contends, inflammatory; or, as Rostan regards it, an affection entirely sui generis; or, as Cruveilhier affirms it to be, capillary apoplexy, and so forth. In the midst of this confusion, Gluge had the great merit of discovering, with the aid of the microscope, a certain test of inflammatory action in many instances of cerebral softening, indicated by the presence in the affected part of exudation corpuscles, or, as they were termed by this writer, "compound inflammation globules." In some valuable papers published in the Edinburgh Medical and Surgical Journal, Dr. Hughes Bennett has shown, by a similar mode of investigation, that two kinds of ramollissement exist, an inflammatory and a non-inflammatory; that these are distinguishable microscopically by plainly recognisable characteristics; and that inflammation in the nervous centres has, by these means, been demonstrated in several instances after it had escaped the search of good morbid anatomists, although indicated by the most unequivocal symptoms. Mr. Solly has added an interesting communication from Dr. Peacock, confirmatory of several of Dr. Bennett's researches, and from which we extract the following conclusions:—

"1st. That in all cases where characteristic symptoms of softening of the brain are present during life, evidences will be found, on microscopic examination, of the extravasation of lymph into the cerebral substance under one or other of the several forms of the so-called exudation granules, corpuscles, or masses:

"2ndly. That the appearance of portions of the brain softened after death, either artificially, by manipulation, or from *post-mortem* change, often, to the naked eye so closely resembles the genuine results of disease as to render it extremely difficult, if not impossible, for practised morbid anatomists to decide between them: and

"3rdly. And consequently that portions of brain, presenting every appearance of softening to the naked eye, but in which the microscope does not reveal the presence of some form of exudation, intermixed with the broken-up cerebral substance, cannot, in the present state of our knowledge, be regarded as having resulted from any diseased process during life." P. 388.

We are happy to find Mr. Solly advocating a principle for which we have always contended, namely, "that every decided deviation from the normal action of the brain is always found to correspond to some alteration of structure." The progress of minute observation, joined to a more just appreciation of the relations existing between the vital actions and the instruments by which they are produced, will doubtless explode the unphilosophical notion that such a thing as a pure functional disease can exist in any part of the body. Lest it should be supposed that this is a mere speculative question in which the practitioner has no concern, we would submit to our readers the following important observations, which coming from a scientific surgeon, who has enjoyed extensive opportunities at Hanwell and elsewhere, of inspecting the brains of lunatics, are deserving of the most serious attention.

"I have long felt convinced," remarks Mr. Solly, "that much of the obscurity which envelopes these diseases and those of other parts of the brain, might be removed by comparing them with diseases of the eye; viewing them through the light which the observation of this interesting class of affections affords us. I do not refer so much to acute disease as chronic, though both are useful as instructors. One great reason why these affections of the eye ought to guide us in our treatment and prognosis of inflammation, both chronic and acute in other organs, is the facility with which we can observe the action of remedies, medicines, topical applications, general stimulants, and diet, upon an organ so open to observation. I believe that every form of mental derangement is dependent on some change, though often very slight and temporary, of the vital condition of the hemispherical ganglion.

"I am convinced that the reason why physicians, to whom the treatment of the insane has been entrusted, believe in the existence of mental disease unattended with disease of the instrument which the mind employs in its communications with the world, is because the medicine, both constitutional and local, has so little controul over these diseases, and the great good to be derived from moral treatment. A knowledge of the treatment of diseases of the eye would teach them a different lesson. Let any man ignorant of the treatment of ophthalmic diseases attempt the cure of a case of strumous ophthalmia; he would, in all probability, seeing the red, inflamed conjunctiva, the pain suffered by the patient, and the distress occasioned by the presence of light, employ all the most approved antiphlogistic measures. He would bleed from the arm, purge violently, and then possibly put his patient under the influence of mercury. What would be the consequence? Why, most assuredly the loss of the eye, total blindness. And the same sad results followed the treatment of insanity when it was considered to be an inflammation of the brain, except in very acute cases occurring in subjects with much constitutional power; injudicious treatment being attended in the one case with the loss of sight, in the other with the loss of intellect. But suppose a judicious surgeon, one bred in the school of Farre, Travers, Lawrence, and my late respected colleague, Frederic Tyrrell, called upon to treat this strumous inflammation of the eye. He would support his patient's general health with a tonic plan of treatment; he would improve the condition of the circulating fluid and the instruments which circulate it. He would endeavour to arrest local inflammation by small local blood-lettings, counter-irritants, and astringent lotions, by removing him from all those atmospheric influences and moral circumstances which would stimulate the organ. And thus he might *ultimately* succeed; but what care, what patience, and what confidence in the remedial agents employed, does it require on the part of the surgeon who treats these cases, to effect a cure!

"If, then, it is so difficult to subdue an inflammation in an organ, the actual condition of whose blood-vessels we can view, to which we can actually apply local remedies, and from which we can withdraw the injurious agents which have produced this inflammation, and exclude the natural stimulus of the organ, and see, in the whole course of our remedial measures, the progress or the failure of each particular plan of treatment, is it astonishing that men should have failed so much in the treatment of chronic and strumous inflammation of the arachnoid, pia mater, and hemispherical ganglion, when they have all these difficulties to contend with, and want many of the adjuncts?" P. 478.

We very much regret that our limits will not allow us to notice the valuable sections treating on apoplexy and convulsive affections; they are eminently of a practical character and merit the careful perusal of every surgeon and physician. For the same reason, we can only briefly refer to the interesting discussion of the author on the nature and treatment of that most intractable affection, epilepsy. As to the seat of the disease, Mr. Solly dissents from the doctrine of Dr. M. Hall, that it is the true spinal cord; being convinced that "the brain and not the spinal cord, is primarily affected." With respect to the pathology of epilepsy, we have the following theory:

"The first morbid action is a sudden determination of blood to the brain, which expends itself, in the secretion of that nervous power which, in a state of health, is employed by the brain to convey volition to the muscles, and which power is, I have no doubt, identical with electricity. This excessive secretion is carried off by the motor nerves, like a discharge from an electric battery, and, from its quantity and excess, produces excessive action of the muscles. It is another illustration of a law that we had occasion to decide upon already, namely, that the first effect of arterial excitement in every secreting organ is to excite to an unnatural degree the natural function of the organ. We know that mental emotion will cause a sudden determination of blood to other organs, which, according to the nature of the part, will be followed or not by secretion.

"Blushing and erection of the penis are instances of sudden determination of blood to a particular part. And the lachrymal glands, salivary glands, testicles, prostate gland, gastric glands, and even the kidneys, often pour forth their secretions so abundantly and so suddenly that the formative fluid, the blood, must have circulated through their capillaries in greater quantity and with greater rapidity than when the glands were at rest, and their secretions suspended. I think that the periodic attacks of mania, with which many of the insane are afflicted, may be regarded in this light." P. 591.

In connexion with this point, the author thus explains his views as to the condition of the blood-vessels in congestion :

"The expression 'determination of blood to the head' is often made use of, but without any explanation of the manner in which this takes place. I doubt whether the profession generally have any distinct idea as to the exact condition of the vascular system which produces it. I would venture to offer the following theory, the first idea of which I certainly derived many years ago from that most truly philosophical work, the Elements of Physics, of Dr. Arnott. It applies not merely to the head, but everywhere else. The middle or muscular coat of the arteries in a state of health contracts with each systole of the ventricles just sufficiently to give a solidity to the wall of the pipe, so that the force of the contraction is not lost on a yielding surface. A much greater force is required to drive water through a leather hose than through a leaden tube. The middle coat contracts just sufficient to assimilate the artery physically and temporarily to the *leaden* tube. Arteries with permanently rigid walls, like leaden tubes, would have interfered by their rigidity with the motions of the limbs; and hence this beautiful contrivance. When this middle coat does not contract, or only contracts imperfectly, then the force of the heart dilates the tubes, and produces congestion.

"I believe then, that determination of blood to the head arises simply from deficient contraction of the muscular coat of the capillaries of the brain, preceded by excitement of the heart's action." P. 592.

The reader will understand from these extracts, that the author regards the essential cause of epilepsy to be a congestion of the arteries and capillaries of the brain, the venous turgescence, which has been noticed by so many writers, being merely the result of the previous arterial accumulation. (p. 595.) In another part of the work, the resemblance of that troublesome affection commonly called 'catchings of the limbs' to epilepsy is pointed out; the cause is thus stated:

"Worry in business, mental anxiety, and vexation of spirit, will sometimes bring on spasmodic action of the muscles, and paralysis. In some cases the anxiety and mental irritation induces disease in the hemispherical ganglion, seriously affecting the temper, but not affecting the intellect. Such cases are familiar to all practical men, but it is very difficult to explain their pathology. I suppose that the disease or diseased action excites unnaturally the tubular neurine, which, commencing in this ganglion as the motor tract, conducts the will to the muscles; and the consequence of this excitement is an irregular supply of stimulus to the muscular system exhibited by the twitchings and spasms. This irregular action the mind can more or less control and arrest when awake; but as soon as sleep takes place, then the spasms commence. I suspect that epilepsy, is a form of this irregular innervation, only that in epilepsy the nervous or electric fluid *accumulates* in undue quantity, and passes off in a large quantity at once, like the discharge of an electric battery. In many cases of epilepsy, the discharge takes place in small quantities both before and after the complete fit. I have two patients under my care now who suffer seriously in this way: one, a single man, has always warning of the advent of the fit by twitchings of the right leg as soon as he drops off to sleep; the other, a married man, has these twitchings so constantly in bed, that his wife is often kept awake during a whole night. In the non-epileptic cases, though electric fluid is secreted in undue quantities, still it does not accumulate, so as to produce a complete convulsive fit, but is constantly oozing out." P. 444.

The fact here noticed that the twitchings usually come on as soon as the person falls asleep, is a strong corroboration of the theory, which attributes all convulsive actions to the true spinal system. We are acquainted with a family, several of the members of which are peculiarly subject to these "catchings," and in all they principally occur during sleep.

But to return from this digression. Mr. Solly subsequently adduces several arguments, to prove that the brain is in a state of congestion during the fit: thus he has always seen a flushing of the face *previous* to the convulsive paroxysm, "previous, as he believes, to the discharge of the electric fluid in those epileptics who were full-blooded and plethoric;" then the amazing benefit he has seen from the sedative influence of digitalis, which medicine is most serviceable when it keeps down the pulse below the standard of health, is another corroboration of the views stated above. Dr. Conolly has also observed that epileptic patients are occasionally warned of the approach of the paroxysm by mental excitement, owing, as Mr. Solly infers, to increased arterial action. Other patients have, as a warning, a singing in the ears, arising it is thought from the dilated carotid vibrating in its osseous canal, like an enlarged artery going to an inflamed part. The evidence of morbid anatomy confirms the idea of this congested state of the blood-vessels, for "in all cases of fatal epilepsy, where there has been an autopsy, the vessels of the brain and membranes have been found enormously distended, and in some there has been extravasation."

"The *Œnanthe crocata*, or hemlock water drop wort, when taken in any quantity, produces epileptic convulsions. I was present at the *post-mortem* examination of four convicts, who died at Woolwich from eating it. The progressive amount of sanguineous effusion on the brain was in proportion to the length of time they survived. The seizure was most striking and instructive.

"In all there was great congestion and some sanguineous effusion on the surface of the brain; in those that lived the longest, the quantity was in proportion to the length of time they survived the seizure. The first man died in about an hour, and the last in about two hours." P. 600.

Among the remote causes of epilepsy it is well to recollect that syphilitic irritation of the dura mater, a fact first pointed out to the author by Mr. Copeland, is to be enumerated.

As regards the treatment of these obstinate affections, Mr. Solly has found the oxide of silver and the continued use of digitalis most efficient as medicines; the latter being adapted to young and excitable subjects in whom it produces the best results, and the former to older patients, where the disease is more confirmed and the fits do not occur so frequently. In order to tranquillize the stomach when the digitalis is administered, creosote or hydrocyanic acid should be given; the digestive organs being generally in fault, will of course require attention. Notwithstanding the conviction the author entertains of the congested state of the cerebral vessels, he does not advocate blood-letting, either general or local.

"I never saw any good derived from blood-letting, and I have seen a *great deal of harm* from it. I bled freely in one or two cases some years ago, under the impression that the disease was inflammatory, when there was a decidedly plethoric state of the system and great congestion of the brain; but I am convinced it caused a repetition of the attacks. Even the application of leeches, either before the attack, at the time, or afterwards, only does harm." P. 615.

There are of course some exceptional cases where depletion may be beneficial; when, for example, there is hypertrophy of the left ventricle, a not unfrequent cause of epilepsy, a few leeches applied to the cardiac region, will be found useful, especially if combined with hydrocyanic acid, which is of great value in subduing irritability of the heart. Foville also recommends the periodical application of leeches to the arms, in plethoric individuals, with large heads, habitually injected with blood.

We must here conclude our notice of this admirable treatise; and, in doing so, we earnestly advise all our professional brethren to enrich their libraries with a work which bears the stamp of extensive observation and careful research; and which has in addition this peculiar advantage, that it combines, in a moderate compass, a scientific and practical account of the anatomy, physiology, and pathology of the brain.

